# Low yield of pathological lymph node metastasis among patients with invasive penile squamous cell carcinoma in the context of high HIV burden: evidence from a prospective cohort study in Zambia

**DOI:** 10.3389/fruro.2026.1727689

**Published:** 2026-02-13

**Authors:** Victor Mapulanga, Owen Ngalamika, Chibamba Mumba, Zoran Muhimbe, Curtis A. Pettaway, Kasonde Bowa, Edford Sinkala

**Affiliations:** 1 Department of Surgery, School of Medicine, University of Zambia, Lusaka, Zambia; 2 Department of Internal Medicine, School of Medicine, University of Zambia, Lusaka, Zambia; 3 Department of Pathology and Microbiology, School of Medicine, University of Zambia, Lusaka, Zambia; 4 M.D Anderson Cancer Center, University of Texas, Houston, TX, United States; 5 School of Medicine and Health Sciences, University of Lusaka, Lusaka, Zambia

**Keywords:** HIV, inguinal lymph node dissection, inguinal lymph node metastasis, pathological yield, penile squamous cell carcinoma

## Abstract

**Introduction:**

Penile squamous cell carcinoma (PSCC) is common in developing countries such as those in sub-Saharan Africa (SSA) and has been attributed to a high prevalence of human papillomavirus (HPV). Additionally, since the prevalence of human immunodeficiency virus (HIV) is high in SSA, and considering that HIV causes reactive lymphadenopathy, this may potentially affect the clinical manifestation, including staging and surgical management, of inguinal lymph nodes in PSCC. Data on surgical staging via inguinal lymph node dissection (ILND) in penile cancer patients from areas of high HIV burden, such as SSA, are scanty. We evaluated the use of ILND as a staging tool to determine the status of inguinal lymph nodes in patients with invasive PSCC in the context of a high HIV burden.

**Methods:**

This was a prospective cross-sectional cohort study of participants recruited between November 2022 and January 2024 at the University Teaching Hospital in Lusaka, Zambia. Patients with surgically resectable PSCC who underwent surgery for both the primary tumor and inguinal lymph nodes simultaneously were recruited into the study. A questionnaire was administered to capture relevant clinical information. The dissected lymph nodes were pathologically analyzed for lymph node number, size, and the presence of metastasis.

**Results:**

Forty patients were enrolled in the study, with a mean age of 53 years (SD 10.28). Thirty-five patients (87.5%) were HIV seropositive, with most patients being virologically suppressed at the time of surgery. Thirty-two patients (80%) presented with clinically palpable inguinal lymph nodes (cN+). The yield of pathological lymph node metastasis (LNM) from surgical staging was 37.5% (12/32) among patients with clinically palpable (cN+) inguinal lymph nodes.

**Conclusion:**

The study demonstrates a modestly low yield of pathological inguinal lymph node metastasis in patients with clinically palpable nodes in the context of a high HIV burden. Minimally invasive biopsy techniques to assess nodal status should be explored in this setting to reduce the morbidity associated with surgical staging while accurately assessing nodal status.

## 1 Introduction

Penile squamous cell carcinoma (PSCC) is rare in developed countries, with incidence rates below 1 per 100,000 in Europe and the United States, accounting for less than 1% of all cancers (1). However, it is more common in developing countries such as those in sub-Saharan Africa (SSA), where the incidence of penile cancer is reported to be as high as 3 per 100,000 (2). This higher incidence in developing countries has partly been attributed to the high prevalence of sexually transmitted infections, such as human papillomavirus (HPV) and human immunodeficiency virus (HIV). Based on the underlying etiology, PSCC is classified as either HPV-associated or HPV-independent, with each having a distinct oncological pathway (3). The prevalence of HPV in PSCC ranges from 22.4% to 66.3% (4), and countries with a high prevalence of HPV have a higher prevalence of HPV-related PSCC. PSCC spreads through the lymphatics, initially to the inguinal lymph nodes and later to the pelvic lymph nodes (5). Inguinal lymph node involvement is the single most important predictor of prognosis and survival in patients with PSCC (6, 7). Curative treatment of PSCC requires treatment of both the primary tumor and inguinal lymph node metastasis. HIV infection, even when controlled with antiretroviral therapy (ART), can cause lymphadenopathy in PSCC patients in the absence of cancer metastases.

Patients with PSCC can present with varying clinical lymph node manifestations, including non-palpable, palpable but mobile, and palpable fixed inguinal lymph nodes. Patients with clinically non-palpable lymph nodes are staged as cN0, while patients with palpable lymph nodes (cN+) are staged as cN1, cN2, or cN3, depending on whether they have single palpable, multiple or bilateral mobile palpable, or palpable fixed nodes, respectively. Patients clinically staged as cN0 have a 20%–25% chance of microscopic metastases of cancer in the lymph nodes, which are discovered in the setting of prophylactic inguinal lymph node dissection (ILND) (8). In contrast, patients with clinically palpable lymph nodes (cN+) have a 45%–80% chance of lymph node metastasis upon ILND (9, 10). There is no reliable noninvasive imaging strategy to determine nodal status, but invasive dynamic sentinel node biopsy (DSNB) has shown higher sensitivity and specificity in the detection of lymph node involvement in PSCC (11). Therefore, lymph node involvement is confirmed on histology following invasive inguinal lymph node dissection (ILND, which carries a high morbidity rate, with complications including skin necrosis, infections, deep vein thrombosis, lymphoedema, and, in some cases, death (12).

Much of the information guiding surgical staging and treatment of inguinal lymph node metastasis is based on HIV-seronegative patients or patients with unknown HIV status. Since HIV infection can cause reactive lymphadenopathy by itself (13), it is unclear how often HIV-positive PSCC patients with clinically palpable lymph nodes turn out to have true inguinal lymph node metastasis and whether all such patients should be subjected to the invasive standard lymph node dissection with its associated complications. Because data regarding inguinal lymph node involvement in penile cancer from HIV-endemic regions such as SSA are scanty, the current study provided an opportunity to further evaluate how clinical and pathological staging of inguinal lymph nodes correlates. Thus, the primary objective of this study was to determine the correlation between clinical inguinal lymph node staging, as assessed by palpation, and pathological outcomes following ILND in a cohort of patients with invasive high-risk PSCC.

## 2 Methods

### 2.1 Study design

#### 2.1.1 Patient selection and clinical staging

This paper focuses on the cross-sectional baseline data of participants recruited into a cohort of PSCC patients between November 2022 and January 2024. This was a service-based cohort study and, given the rarity of penile cancer, sample size was determined by the number of eligible patients who presented to the dedicated high-volume penile cancer unit after its establishment. During the study period, 56 patients with PSCC were seen, and only 40 met the inclusion criteria. Eligible participants were patients with histologically confirmed PSCC based on diagnostic wedge biopsy of the primary tumor. Participants with clinically non-palpable inguinal lymph nodes (cN0) were included in the study if the diagnostic tumor biopsy revealed moderate or poorly differentiated tumors (i.e., grades 2–3) or exhibited involvement of the corpora spongiosum or cavernosum (i.e., pT2–pT3). Patients with well-differentiated (i.e., grade 1), low-stage tumors (i.e., ≤T1) and non-palpable inguinal lymph nodes were not included in the study, as they did not undergo inguinal lymph node dissection. Further, patients had to be amenable to primary tumor resection and inguinal lymph node resection in the same operative setting. Due to limited access to computed tomography scans at the time of the study, patients were staged via inguinal palpation alone, without additional cross-sectional imaging. Patients with lymph node masses that were not surgically resectable were excluded from the study. Consenting patients scheduled for surgical treatment were recruited, with sociodemographic and clinical information collected using a questionnaire prior to surgery. ILND of the femoral triangle was performed, with the inguinal ligament superiorly, the adductor longus muscle medially, and the sartorius muscle laterally forming its margins.

#### 2.1.2 Inguinal surgery

Participants with palpable inguinal lymph nodes underwent standard inguinal lymph node dissection (ILND) of the femoral triangle, whose margins were the inguinal ligament superiorly, the adductor longus muscle medially, and the sartorius muscle laterally (14).

Participants with non-palpable inguinal lymph nodes underwent a modified inguinal lymph node dissection (mILND), with a comparatively smaller incision and dissection, preserving the area lateral to the femoral artery and the saphenous vein (15). Dynamic sentinel lymph node biopsy was unavailable due to limited resources and training.

### 2.2 Histopathology procedure

Resected PSCC tumors and dissected inguinal lymph nodes were placed in 10% neutral-buffered formalin and immediately transported from the operating theater to the pathology laboratory following the procedure. At the pathology laboratory, gross descriptions of the tumors were made before fixation in 10% neutral-buffered formalin, followed by paraffin embedding. Histopathological analysis was performed after hematoxylin and eosin staining was done, followed by p16 staining by immunohistochemistry as a surrogate marker for HPV presence in the tumor (16).

### 2.3 Statistical analysis

The primary outcome variable was the incidence of histologically determined inguinal lymph node metastasis in the study cohort. This was a binary variable coded as either positive or negative. Predictor variables for lymph node metastasis were tested for normality using the Shapiro–Wilk normality test. A Student’s t-test was used to compare normally distributed continuous data between groups, while the Mann–Whitney U test was used for non-normally distributed continuous variables. Fisher’s exact test or the chi-square test was used for comparisons between binary outcome and binary categorical variables. A p-value of <0.05 was considered statistically significant. Stata version 17 was used for data analysis.

### 2.4 Ethical consideration

Written informed consent was obtained from all participants after providing information about the study. Confidentiality was maintained throughout data collection and analysis. Ethical clearance was obtained from the University of Zambia Biomedical and Research Ethics Committee (REF 3233-2022), and authority to conduct the study was obtained from the National Health Research Authority.

## 3 Results

### 3.1 Demographic and clinical characteristics

A total of 40 participants were enrolled in the study. The mean age was 53 years (SD 10.28), with an age range of 29–74 years. Of the 40 participants, 35 (87.5%) were HIV seropositive and had been on antiretroviral therapy for a minimum of 2 years at the time of surgical treatment. Among the enrolled participants, 39 had their HPV status established using p16 immunohistochemistry, with an HPV prevalence of 67%. The median CD4 count among HIV-positive participants was 464 cells/µL. The median duration of penile symptoms among the participants was 8 months, and the mean size of the primary tumor was 43 mm. **Table 1** shows the clinical and demographic characteristics of the study participants.

**Table 1:** Demographic and clinical characteristics of the participants.

Variable	Minimum	Maximum	Mean	Median	Proportion n (%)
Age (years)	29	74	54	54.5	
Duration of symptoms (months)	3	90	15	8	
Tumor size (mm)	4	79	43	45	
Number of lymph nodes retrieved per groin (n = 80)					
cN0 (n = 16)	4	15	6.9		
cN+ (n = 64)	3	22	8.2		
HIV status					
Positive					35 (87.5%)
Negative					5 (12.5%)
CD4 count (cells/µL)	155	1,632	537	464	
HPV status					
Positive					26 (67%)
Negative					13 (33%)

**Table 1**. Demographic and clinical characteristics of the participants.

### 3.2 Correlation between clinical and histological characteristics

Of the 40 participants, 32 (80%) presented with clinically palpable inguinal lymph nodes (cN1, cN2, or cN3), of whom only 12/32 (37.5%) had histologically confirmed inguinal lymph node metastasis. The breakdown is shown in **Table 2**.

**Table 2:** Comparison of the clinical status and the pathological status of lymph nodes.

Clinical stage of lymph nodes	Total (n)	Lymph node negative (pN0)	Lymph node positive (pN+)
cN0	8	8	0
cN1	1	1	0
cN2	29	19	10
cN3	2	0	2
cN+	32	20	12
Total	40 (100)	28	12

*cN+ = cN1 + cN2 + cN3.

**Table 2**. Comparison of the clinical status with the pathological status of lymph nodes.

**Figure 1:**
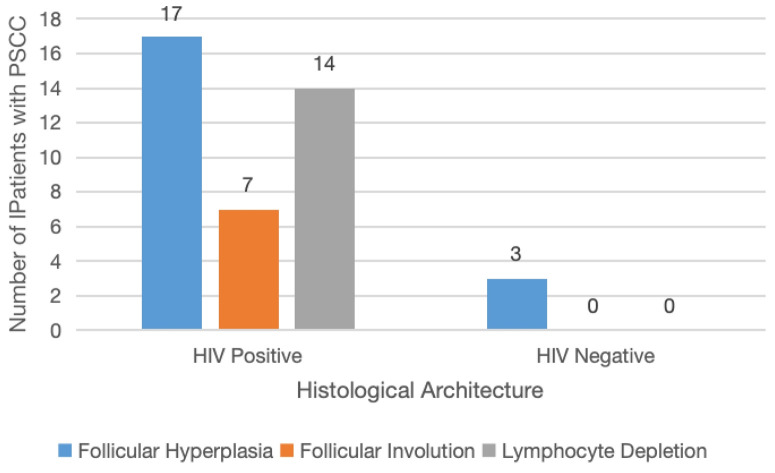
Histological images of lymph nodes for both metastatic and non-metastatic inguinal lymph nodes.

The majority of the 20 lymph nodes that were negative for metastasis on histology demonstrated reactive lymphadenopathy (**Figure 1**). The presence of lymph node metastases is also shown in **Figure 1**.

**Figure 2:**
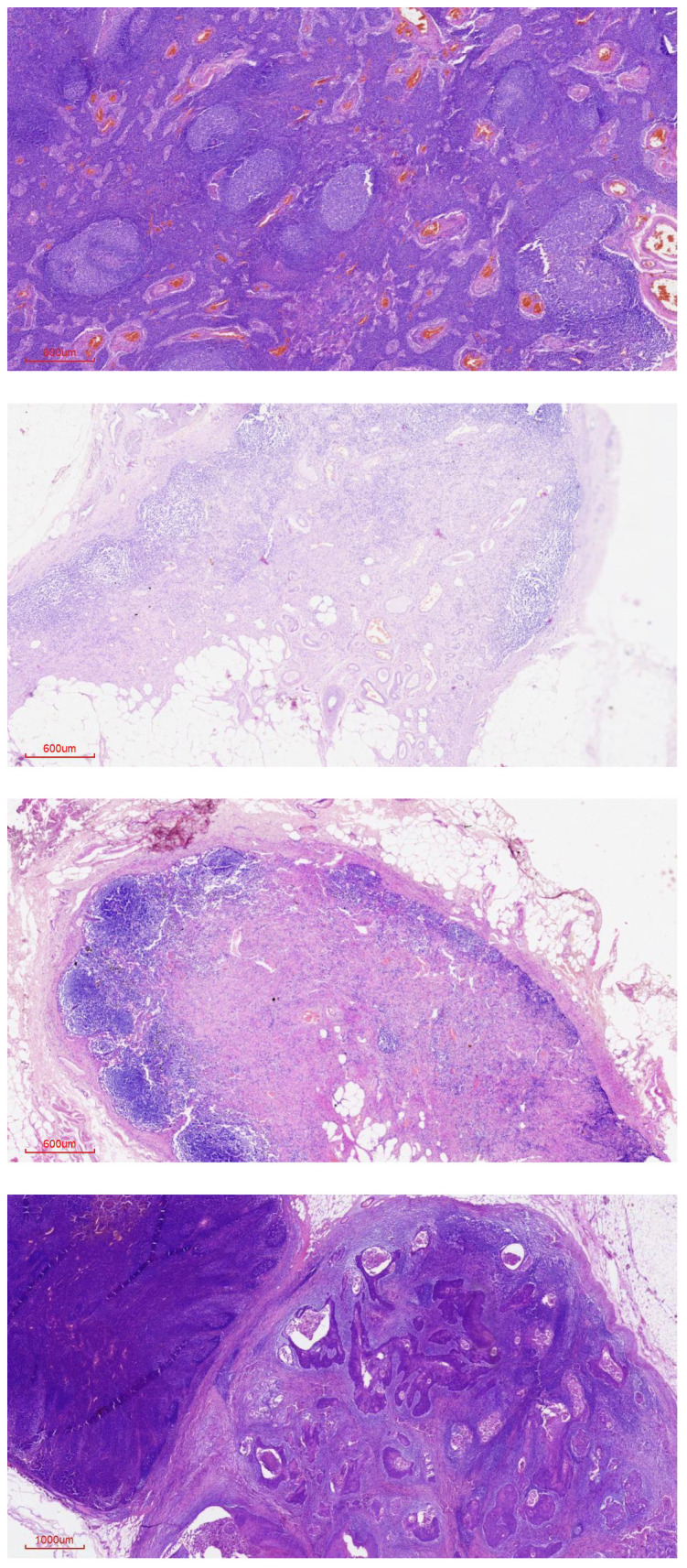
Reactive lymph node histological architecture in HIV-positive and HIV-negative participants.

Reactive lymphadenopathy demonstrated follicular hyperplasia in HIV-negative patients, whereas in HIV-positive patients, the histological architecture ranged from follicular hyperplasia to lymphocyte depletion, as shown in **Figure 2**.

### 3.3 Associations between pathological lymph node status and predictor variables

**Table 3** shows the associations between the pathological lymph node status and the predictor variables. Variables associated with pathological inguinal lymph node involvement included younger age (p = 0.04), advanced pathological stage of the primary tumor (p < 0.05), and perineural involvement (p = 0.03). On multivariate logistic regression analysis, only pathological stage remained an independent predictor of lymph node metastasis (p < 0.05).

**Table 3:** Associations between the pathological lymph node status and predictor variables.

	Lymph node negative	Lymph node positive	p-value
Age in years (mean)	56	49	0.04
Duration of symptoms in months (median)	8	5	1.00
HIV status (n)			0.47
Negative	3	2	
Positive	25	10	
HPV status			0.12
Negative	11	2	
Positive	17	9	
Tumor differentiation (n)			1.00
Mild	6	2	
Moderate	14	7	
Poorly	8	3	
Tumor size in mm (mean)	42	45	0.63
Pathological stage (n)			0.001
pT1	1	0	
pT2	25	0	
pT3	0	8	
Perineural involvement (n)			0.03
Absent	15		
Present	13	2	
Lymph vascular involvement (n)			0.48
Absent	2	0	
Present	26	12	

**Table 3**. Associations between pathological lymph node status and predictor variables.

## 4 Discussion

Our study has demonstrated a relatively low positive yield of pathological inguinal lymph node metastasis among dissected inguinal lymph nodes in patients with PSCC compared with other studies (10). Among the PSCC patients with clinically palpable inguinal lymph nodes, only 37.5% were confirmed to have lymph node metastasis on histology, while the remainder had reactive adenopathy. The histological architecture of reactive lymphadenopathy differed between HIV-positive and HIV-negative patients. HIV-positive patients demonstrated a spectrum ranging from follicular hyperplasia to follicular involution and lymphocyte depletion, whereas HIV-negative patients demonstrated follicular hyperplasia only. This finding is consistent with studies of reactive lymphadenopathy showing lymphocyte depletion in HIV-infected patients (17). The relatively low yield of pathological inguinal lymph node metastasis observed in patients with clinically palpable inguinal lymph nodes contrasts with reports indicating up to an 80% prevalence of lymph node metastasis in such patients with PSCC (9, 10). However, our findings are comparable with a study from South Africa—a country within the same region and with a similarly high HIV prevalence among PSCC patients (78.1%)—which reported a similarly low proportion of histologically confirmed lymph node metastasis (43.4%) despite a high proportion of individuals presenting with clinically palpable lymph nodes (18). The low prevalence of pathological lymph node metastasis observed in our study may be partly explained by the high prevalence of HIV infection (87.5%) among our PSCC participants, as HIV itself is associated with reactive lymphadenopathy rather than malignant lymph node involvement. The prevalence of HIV among PSCC patients in this study was substantially higher than the national HIV prevalence of 11% (19). This high HIV prevalence is comparable to that reported in PSCC patients compared with South Africa, another country in SSA, where an HIV prevalence of 84% was reported among patients diagnosed with PSCC (18). All HIV-seropositive participants in our study had been diagnosed with HIV at least 2 years prior to enrollment and had no clinical features of advanced HIV disease. This is reflected in the relatively high median CD4 count of 464 cells/µL and the fact that all HIV-seropositive participants were virologically suppressed at the time of the study. Despite viral suppression, the high HIV prevalence among PSCC patients supports the classification of PSCC as being an HIV-associated, but not AIDS-defining, malignancy (20, 21).

Even though the HIV-seropositive participants in this study were virologically suppressed, they still developed penile cancer. This may be attributed to delayed initiation of antiretroviral therapy, whereby immune reconstitution primarily restores CD4 T-cell counts without fully restoring CD8 T-cell–mediated immune surveillance, potentially leaving the individuals immunologically vulnerable to fight cancer initiation and progression.

This study demonstrated that the mean age at presentation with PSCC in the context of a high HIV burden was lower than that reported in studies from other settings (22). The mean age of patients with PSCC in our cohort was 53 years, with the youngest patient aged 29 years and the oldest aged 74 years. This contrasts with developed countries such as the United States, where the median age at diagnosis of PSCC has been reported as 68 years in the general population and 62 years in the Black population (22). Similarly, studies from European countries such as Germany report a mean age at diagnosis of 70 years (23), which is considerably higher than what we observed. Our findings are comparable with those reported from other African countries, including Mozambique (mean age at presentation of 50.9 years), Tanzania (median age at presentation of 47 years), and South Africa (mean age at presentation of 50 years) (24–26). The difference between European and African populations may be related to the higher prevalence of HPV-associated PSCC in our African settings.

In this study, the prevalence of HPV among participants was 67% (26/39), which is higher than that reported in other African countries, such as Rwanda (20%), Guinea (16.7%), and South Africa (41.5%) (27). This higher prevalence may reflect differences in the prevalence of sexually transmitted infections, variation in penile cancer subtypes, or the use of robust HPV testing methods in this study. We found no significant association between HPV status and inguinal lymph node involvement, consistent with other studies reporting that HPV is not protective against lymph node metastasis (LNM) (28). However, some studies have reported a statistically significant protective effect of HPV against inguinal LNM (29).

Lymph node metastasis is the single most important predictor of prognosis in patients with PSCC. Therefore, identifying predictors of LNM is critical for patient management. In this study, younger age, perineural invasion, and advanced pathological tumor stage were associated with inguinal LNM. In contrast, HIV status, HPV status, tumor differentiation, and lymphovascular invasion were not predictive of inguinal LNM. This study also highlights the feasibility and benefits of centralizing penile cancer care. All participants were treated at a high-volume specialized center developed, in part for this purpose. Patients received not only treatment of the primary tumor but also appropriate surgical evaluation and management of inguinal lymph nodes in accordance with current penile cancer guidelines (30). This is noteworthy, as guideline-based inguinal lymph node staging has been associated with favorable outcomes but is sometimes neglected in decentralized care settings (31, 32).

Several limitations should be considered. Preoperative lymph node size and extent could not be assessed using imaging, as clinical staging was based on palpation alone. This may have resulted in understaging in some cases. However, this limitation was unavoidable due to restricted access to imaging facilities during the study period. The availability of computed tomography scanning would have enabled correlation between preoperative lymph node size and pathologically confirmed LNM, as well as detection of occult metastatic disease. In addition, the study population was limited to patients with surgically resectable lymph nodes who were scheduled for surgical treatment. The cohort also consisted predominantly of HIV-infected participants on antiretroviral therapy with suppressed viral loads, limiting the ability to assess the impact of uncontrolled HIV infection. Furthermore, the number of HIV-negative participants was too small to allow meaningful statistical comparison of reactive lymphadenopathy architecture between HIV-positive and HIV-negative groups. Despite these limitations, the prospective study design, high HIV prevalence, high-volume study site, and the study findings provide a unique contribution to the existing literature.

## 5 Conclusion

Our findings indicate that the use of inguinal lymph node dissection for staging of inguinal lymph nodes in patients with PSCC in the context of a high HIV burden yields a relatively low rate of pathologically positive lymph node involvement. Less invasive methods of lymph node staging approaches, such as open lymph node biopsy in patients with clinically palpable lymph nodes and dynamic sentinel node biopsy in patients with clinically negative lymph nodes, may have a role in this setting. Further prospective studies in high HIV-endemic regions are needed to validate these findings.

## Data Availability

The raw data supporting the conclusions of this article will be made available by the authors without undue reservation.
